# Median Nerve Entrapment by Superficial Brachioulnar Artery: Anatomical Insights and Clinical Perspective

**DOI:** 10.7759/cureus.66228

**Published:** 2024-08-05

**Authors:** Parul Kaushal, Jessy J P, Arthi Ganapathy

**Affiliations:** 1 Anatomy, All India Institute of Medical Sciences, New Delhi, New Delhi, IND

**Keywords:** neurovascular compression, entrapment neuropathy, anatomical variation, superficial brachioulnar artery, median nerve compression

## Abstract

Entrapment neuropathy of the median nerve is typically caused by compression at specific anatomical points. However, idiopathic cases, where the standard anatomical compression points are normal, pose diagnostic challenges. This report highlights a unique case discovered during an anatomical dissection of the right upper limb in a 62-year-old male cadaver, where the median nerve was compressed by an unusual branch of the brachial artery, termed the superficial brachioulnar artery (SBUA). The median nerve formed at the distal half of the arm, receiving additional components from the lateral cord, with a noted communication with the musculocutaneous nerve. The SBUA, originating from the brachial artery, passed between the roots of the median nerve and continued superficially, forming the superficial palmar arch. The coexistence of neurovascular variations is clinically significant as it may lead to nerve compression and subsequent symptoms. This case is the first documented instance of median nerve compression by an SBUA. Such variations are crucial for surgical and diagnostic procedures, as abnormal vascular structures can be mistaken for veins, leading to iatrogenic injuries. In addition, understanding these variations helps explain idiopathic median nerve neuropathies and highlights the need for thorough anatomical knowledge to prevent complications during surgical interventions.

## Introduction

The intricate network of somatic nerves in the upper and lower limbs is susceptible to entrapment neuropathy, a condition characterized by compression, either at the point of entry into a muscle or due to the pressure exerted by a fibrous band [1]. Among these nerves, the median nerve, often referred to as the "eye of the hand," holds pivotal significance for hand functionality. Responsible for innervating the flexor-pronator muscles in the forearm and various muscles in the radial part of the hand, the median nerve plays a vital role in controlling an array of hand movements. In addition to its motor functions, the nerve provides sensory innervation to specific areas on the palmar and dorsal surfaces of the thumb, fingers, and hand [2].

While established causes of median nerve entrapment include compression at various anatomical points, such as between the two heads of pronator teres, under the flexor retinaculum (3% of the general population) (leading to carpal tunnel syndrome), and rarely (0.5%) at the distal humerus by the ligament of Struthers or proximal elbow by a thickened biceps aponeurosis, there remains a subset of cases categorized as idiopathic. These instances pose diagnostic challenges, particularly when nerve compression presents without motor paralysis. The difficulties of diagnosing median nerve compression without motor paralysis imply the need to explore potential unusual sites of nerve compression. Such atypical locations may arise due to variations in the formation and course of the nerve or variations in vascular structures closely associated with the nerve [3].

Understanding the anatomical course of the median nerve is crucial in diagnosing these idiopathic causes. Originating in the axilla, adjacent to the axillary artery, the median nerve forms through the union of its medial and lateral roots from the brachial plexus. The lateral root follows a superficial course to the third part of the axillary artery, crossing to the medial aspect to join the medial root, forming the median nerve lateral to the axillary artery. Descending from the axilla, the median nerve enters the arm adjacent to the brachial artery, gradually crossing anterior to the artery to come to lie medial to it in the cubital fossa. A consistent anatomical relationship with the brachial artery is observed throughout its course [4].

As the median nerve progresses distally, it crosses to the medial side of the brachial artery superficially and assumes an anterior position relative to the elbow joint. As the median nerve is closely related to the brachial artery and crosses it superficially, any variation in the branches of the artery crossing over the median nerve could form a potential source of nerve compression [5-6]. Here, we report a case of an unusual branch of the brachial artery (superficial brachioulnar artery (SBUA)) crossing over between the median and lateral roots of the median nerve formed at a much lower level than its usual site.

## Case presentation

**Figure 1:**
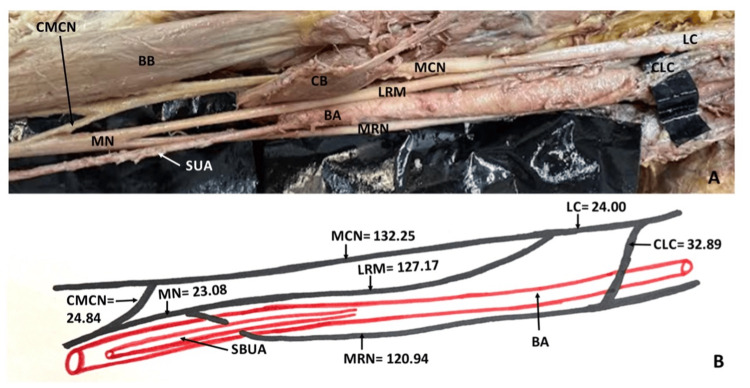
Photograph of the dissected right upper limb and schematic representation of the observed variations (A): Low formation of the median nerve, below the insertion of the coracobrachialis and demonstrating the anatomical relationship between the SBUA and median nerve. Note the communicating branches with the lateral cord and the musculocutaneous nerve. Superficial brachioulnar artery is seen passing between the loop of formation of the median nerve. (B) Schematic diagram showing the measurements (mm) of various nerve components and the anatomical relationship between the SBUA and median nerve. Figure abbreviations: median nerve (MN); lateral root of median nerve (LRM); lateral cord of brachial plexus (LC); medial root of median nerve (MRN); communication between the lateral cord and the medial root of the median nerve (CLC); communication between the musculocutaneous nerve and the median nerve (CMCN); superficial ulnar artery (SUA); brachial artery (BA) Figure 1B credits: PK (author)

During educational gross anatomy dissection at the Department of Anatomy, we came across a variant neurovascular pattern in the right upper limb of a 62-year-old embalmed male cadaver. The median nerve was formed in the distal half of the arm by the union of the lateral and medial roots coming from the lateral and medial cords, respectively. The medial root of the median nerve received an additional component from the lateral cord; this communicating branch (length = 32.89 mm) crossed superficially to the artery. Furthermore, distally, in the course of the median nerve (23.08 mm distal to its formation), a communicating branch (length = 24.84 mm) was noted. This communicating branch joined the musculocutaneous nerve just below the insertion of the coracobrachialis muscle (132.25 mm distal to the origin of the nerve) (Figure 1A, 1B). Besides the low formation of the median nerve and its unusual communications with the neighboring nerves, it was interesting to see a branch of the brachial artery passing between the loop formed by two roots of the median nerve. This branch was identified as the ulnar artery having a high origin from the brachial artery. When traced distally, this artery passed superficial to all the flexor forearm muscles except palmaris brevis, continued into the palm, and formed the superficial palmar arch. Hence, it was called the SBUA. The brachial artery was divided into radial and common interosseous arteries, which followed their usual course. The institutional guidelines for the procurement of human cadavers and their use for medical teaching and research were strictly adhered to. The anonymized body donor included in this study gave informed consent during his lifetime to be part of research projects and student training. The ethics committee declared the use of these corpses for scientific studies as legal. Therefore, no separate vote of the local ethics committee was required.

## Discussion

While isolated cases of anatomical variations in the nervous and arterial structures of the upper limb are common, the coexistence of neurovascular variations along the upper limb is rare and clinically relevant as abnormal vascular structures can compress the nerves and produce symptoms. Compression of the median nerve by the superficial brachial artery has been previously reported [6]. However, this is the first documented case of median nerve compression by an SBUA.

The SBUA refers to the ulnar artery of high origin, which runs superficial to the forearm muscles except the palmaris longus. This rare artery with a reported incidence of ~3% [7] usually arises from the axillary [5] or the brachial artery [8]. Owing to its superficial location, the SBUA is at high risk of iatrogenic injuries being mistaken for a superficial vein. Intra-arterial injections and cannulation may cause ischemia of the upper extremity [9]. Furthermore, harvesting the radial artery for coronary artery bypass surgeries in such cases leaves SBUA as the only source of blood to the distal parts of the upper limb. The presence of two arteries in the arm results in complicated blood pressure readings. Moreover, its perils, SBUA, is of great significance during free radical forearm flap creation [10]. Embryologically, the presence of this artery is explained by the hemodynamic persistence of the superficial system over the deep system during the fifth stage of the development of arteries of the upper limb, as the ulnar artery branches from the brachial artery during the third stage and later regresses [11].

The loop of formation of the median nerve is placed in the distal half of the humeral length in ~4% of cases [1,12] and was noted in the present study. The formation of the median nerve by multiple roots has been documented by earlier investigators [13], and the communicating branch between the lateral cord and medial root of the median nerve, as observed in the present study, is the most reported pattern [14]. A communication between the median and musculocutaneous nerve, corresponding to pattern 2 [15] and type II [16], was noted in the present case. Variant nerves with abnormal origin and communications are usually more prone to accidental injuries and entrapment neuropathies [17]. The knowledge of communicating branches helps to explain unusual clinical signs and causes of idiopathic median nerve neuropathies. Saeed and Rufai [18] have suggested that nerve fibers from the fifth and sixth cervical ventral rami get hitched along the median nerve by its variant lateral root and join the musculocutaneous nerve via the communicating branch. Another theory about the communication between the two nerves is that the musculocutaneous nerve is derived from the median nerve during embryonic development and any deviation in the development may result in the persistence of such embryonic patterns [19].

The coexistence of neurovascular variations highlights the crucial role played by molecules, such as cadherins and ephrins, which are common to the development of both these components. Furthermore, the developing axons are guided by the synchronized and region-specific expression of various chemo-attractants and chemo-repellents, and any alteration of this precise mechanism can lead to variant neuronal patterns [20]. Simultaneous occurrence of arterial deviations with variations in the brachial plexus may result in an incomplete block of the plexus [12]. In addition, the long-standing compression of the median nerve results in neuropathies and muscle weakness, while the involvement of vascular structure results in claudicating pain in the upper limb, a combination of symptoms that perplex clinicians. The presence of the SBUA can lead to misdiagnosis of median nerve entrapment syndromes, given its potential to mimic other common entrapment sites.

## Conclusions

The vast variant anatomy exhibited by the brachial plexus and the vascular structures around it mandates prior acquaintance of surgeons and interventional radiologists with the co-existence of a plethora of arterial and nervous anomalies, to avoid complications during various surgical procedures in the region. This case marks the first documented instance of the SBUA passing between the two roots of the median nerve, which might result in the compression of the median nerve by the SBUA or vice versa. Prior knowledge of such cases where the coexistence of neural and vascular symptoms may jeopardize the clinicians is crucial in explaining long-standing idiopathic median nerve neuropathies and muscle numbness contemporaneous with claudicating pain in the upper limb due to compromised blood supply. Acquaintance with the variant patterns of communicating branches of the median nerve with its neighboring nerves helps in reducing the rate of failure of nerve blocks in the region of the axilla. Awareness of the existence of the SBUA is critical as the most common complications associated with it are inadvertent intra-arterial injections and errors in blood pressure readings, which are routinely conducted procedures. Furthermore, this study underscores the necessity of thorough evaluation of individual cases prior to any surgical intervention to prevent complications during or after the procedure. This study also opens avenues for further research in deciphering the molecular factors involved in the development of neural and arterial structures.

## References

[1] Wozniak J, Kedzia A, Dudek K (2012). Anatomical variability of median nerve formation in human foetuses in clinical aspect. Adv Clin Exp Med.

[2] Drake R, Vogl W, Mitchell W (2015). Gray's anatomy for students. https://books.google.com/books?hl=en&lr=&id=_ozrqnzzhFwC&oi=fnd&pg=PP1&dq=Gray%27s+Anatomy+for+Students+Philadelphia&ots=DU6NEefNEC&sig=0Df-JUQcC5vC9MgFg0HAIgeHZwc.

[3] Miller TT, Reinus WR (2010). Nerve entrapment syndromes of the elbow, forearm, and wrist. AJR Am J Roentgenol.

[4] El-Haj M, Ding W, Sharma K, Novak C, Mackinnon SE, Patterson JM (2021). Median nerve compression in the forearm: a clinical diagnosis. Hand (N Y).

[5] Natsis K, Papadopoulou AL, Paraskevas G, Totlis T, Tsikaras P (2006). High origin of a superficial ulnar artery arising from the axillary artery: anatomy, embryology, clinical significance and a review of the literature. Folia Morphol (Warsz).

[6] Nkomozepi P, Xhakaza N, Swanepoel E (2017). Superficial brachial artery: a possible cause for idiopathic median nerve entrapment neuropathy. Folia Morphol (Warsz).

[7] Rodríguez-Niedenführ M, Vázquez T, Nearn L, Ferreira B, Parkin I, Sañudo JR (2001). Variations of the arterial pattern in the upper limb revisited: a morphological and statistical study, with a review of the literature. J Anat.

[8] Sieger J, Patel L, Sheikh K, Parker E, Sheng M, Sakthi-Velavan S (2019). Superficial brachioulnar artery and its clinical significance. Anat Cell Biol.

[9] Chin KJ, Singh K (2005). The superficial ulnar artery--a potential hazard in patients with difficult venous access. Br J Anaesth.

[10] Casal D, Pais D, Toscano T (2012). A rare variant of the ulnar artery with important clinical implications: a case report. BMC Res Notes.

[11] Singer E (1933). Embryological pattern persisting in the arteries of the arm. Anat Rec.

[12] Claassen H, Schmitt O, Wree A, Schulze M (2016). Variations in brachial plexus with respect to concomitant accompanying aberrant arm arteries. Ann Anat.

[13] Satyanaraya N, Vishvakarma N, Kumar G, Guha R, Datta A, Sunitha P (2009). Rare variations in the formation of median nerve-embryological basis. Nepal Med Col J.

[14] Song ZF, Sun MM, Wu ZY, Lv HZ, Xia CL (2014). Anatomical study of the communicating branches of cords of the brachial plexus and their clinical implications. Clin Anat.

[15] Choi D, Rodríguez-Niedenführ M, Vázquez T, Parkin I, Sañudo JR (2002). Patterns of connections between the musculocutaneous and median nerves in the axilla and arm. Clin Anat.

[16] Venieratos D, Anagnostopoulou S (199811). Classification of communications between the musculocutaneous and median nerves. Clin Anat.

[17] Roberts W (1992). Anomalous course of the median nerve medial to the trochlea and anterior to the median epicondyle of the humerus. Anat Anz.

[18] Saeed M, Rufai AA (2003). Median and musculocutaneous nerves: variant formation and distribution. Clin Anat.

[19] Mahan M, Spinner RJ (2016). Nerves of the upper extremity. Bergman's Comprehensive Encyclopedia of Human Anatomic Variation.

[20] Carmeliet P (2003). Blood vessels and nerves: common signals, pathways and diseases. Nat Rev Genet.

